# Towards Environmental RF-EMF Assessment of mmWave High-Node Density Complex Heterogeneous Environments

**DOI:** 10.3390/s21248419

**Published:** 2021-12-16

**Authors:** Mikel Celaya-Echarri, Leyre Azpilicueta, Fidel Alejandro Rodríguez-Corbo, Peio Lopez-Iturri, Victoria Ramos, Mohammad Alibakhshikenari, Raed M. Shubair, Francisco Falcone

**Affiliations:** 1 School of Engineering and Sciences, Tecnologico de Monterrey, Monterrey 64849, Mexico; mikelcelaya@tec.mx (M.C.-E.); fidel.rodriguez@tec.mx (F.A.R.-C.); 2 Department of Electric, Electronic and Communication Engineering, Public University of Navarre, 31006 Pamplona, Spain; peio.lopez@unavarra.es (P.L.-I.); francisco.falcone@unavarra.es (F.F.); 3 Institute of Smart Cities, Public University of Navarre, 31006 Pamplona, Spain; 4 Telemedicine and Digital Health Research Unit, Health Institute Carlos III, 28029 Madrid, Spain; vramos@isciii.es; 5 Department of Signal Theory and Communications, Universidad Carlos III de Madrid, 28911 Leganés, Madrid, Spain; mohammad.alibakhshikenari@uc3m.es; 6 Department of Electrical and Computer Engineering, New York University (NYU) Abu Dhabi, Abu Dhabi 129188, United Arab Emirates; raed.shubair@nyu.edu

**Keywords:** radiofrequency electromagnetic fields (RF-EMF), mmWave, electromagnetic safety, 3D ray launching (3D-RL), 5G, 802.11ay, propagation modeling

## Abstract

The densification of multiple wireless communication systems that coexist nowadays, as well as the 5G new generation cellular systems advent towards the millimeter wave (mmWave) frequency range, give rise to complex context-aware scenarios with high-node density heterogeneous networks. In this work, a radiofrequency electromagnetic field (RF-EMF) exposure assessment from an empirical and modeling approach for a large, complex indoor setting with high node density and traffic is presented. For that purpose, an intensive and comprehensive in-depth RF-EMF E-field characterization study is provided in a public library study case, considering dense personal mobile communications (5G FR2 @28 GHz) and wireless 802.11ay (@60 GHz) data access services on the mmWave frequency range. By means of an enhanced in-house deterministic 3D ray launching (3D-RL) simulation tool for RF-EMF exposure assessment, different complex heterogenous scenarios of high complexity are assessed in realistic operation conditions, considering different user distributions and densities. The use of directive antennas and MIMO beamforming techniques, as well as all the corresponding features in terms of radio wave propagation, such as the body shielding effect, dispersive material properties of obstacles, the impact of the distribution of scatterers and the associated electromagnetic propagation phenomena, are considered for simulation. Discussion regarding the contribution and impact of the coexistence of multiple heterogeneous networks and services is presented, verifying compliance with the current established international regulation limits with exposure levels far below the aforementioned limits. Finally, the proposed simulation technique is validated with a complete empirical campaign of measurements, showing good agreement. In consequence, the obtained datasets and simulation estimations, along with the proposed RF-EMF simulation tool, could be a reference approach for the design, deployment and exposure assessment of the current and future wireless communication technologies on the mmWave spectrum, where massive high-node density heterogeneous networks are expected.

## 1. Introduction

A step forward toward the use of millimeter waves (mmWaves) in fifth-generation (5G) radio interface technology allows the use of larger bandwidth than previous mobile generations, allowing the possibility to deliver gigabit per second (Gb/s) wireless services. This significant increase in traffic data has been conceived to cover multiple usage scenarios, from enhanced mobile broadband to ultra-reliable low-latency communications (URLLC), going through massive Internet of Things (IoT) connections. New standards, such as the 5G New Radio (5G-NR) [1,2,3], have included innovative techniques and procedures to overcome the unique challenges associated with mmWave transmissions. For example, mmWave transmitter antennas must be directional to take advantage of beamforming gains and cope with increased path loss and other propagation losses compared to sub-6 GHz frequency bands. Besides, 5G-NR benefits from a high flexibility level in several domains, such as the time domain (i.e., variable Time Division Duplex (TDD) schemes), frequency domain (i.e., bandwidth fractions), spatial domain (i.e., high adaptability in the implementation of beam sweeping or Multi-User Multiple Input Multiple Output (MU-MIMO) technology) and scalable numerology (enabling variations in subcarrier spacing as a function of the numerology parameter µ ranging from 0 to 5 from 15 kHz to 480 kHz and slot lengths given by 1/2 µ ms) in order to optimize the usage of temporal and spatial resources in the communication channel.

For indoor scenarios with a high user density (i.e., convention centers, event halls, concerts, indoor stadiums, etc.) and/or enterprise deployments (i.e., office buildings, shop floors, meeting rooms, auditoriums, libraries, etc.), 5G-NR mmWave can complement existing Wireless Local Area Network (WLAN) deployments with new and enhanced mobile broadband experiences, bringing a multi-Gb/s low-latency channel capacity, supporting devices beyond smartphones (i.e., tablets, always-connected laptops, augmented reality (AR)/virtual reality (VR), etc.) and therefore leveraging the existing infrastructure.

The advantages of 5G networks are well-discussed in the literature [4,5], and there is no doubt about the need for faster and more reliable wireless communication system deployments, with broadband data access in crowded locations. However, at the same time, the implementation of mmWave new technologies has increased the population concern over the possible impact on health and safety arising from the radiated electromagnetic field (EMF) exposure by these systems. This concern has led to the requirement of having accurate EMF simulation and measurement techniques to analyze the radiation exposure in the current and future wireless crowded scenarios. These techniques can verify compliance or not with the regulations from the point of view of radioelectric exposure of nonionizing radiation.

In the past few years, there has been a significant effort by the research community to provide clear EMF exposure insight with the presentation of different models for the RF-EMF assessment of 5G communication systems. From an engineering perspective, these RF-EMF exposure assessment models can aid in the design and deployment of 5G communication systems, achieving a good tradeoff between efficiency and operation, minimizing radiation exposure. On the one hand, most of the works are carried out in outdoor scenarios and present EMF assessments focused on the downlink (DL) of a 5G system. The work presented in Reference [6] proposed a simulation technique to assess EMF exposure in 5G cellular systems operating at sub-6 GHz frequencies. The novelty of their work is that they propose a localization-enhanced pencil beamforming technique in which the traffic beams are tuned in accordance with the uncertainty localization levels of the User Equipment (UE) in the DL configuration. The authors in Reference [7] presented a computational method based on ray tracing techniques (RT) to estimate human EMF exposure in DL 5G base stations at sub-6 GHz frequencies in outdoor macrocell environments. The same authors presented in References [8,9,10] a combined numerical approach based on RT and Finite Difference Time Domain (FDTD) techniques, which estimates EMF exposure for 5G massive MIMO in different environments, such as an industrial environment [8], an urban microenvironment (UMi) [9] or an urban macro-environment (UMa) [10], all of them at 5G frequencies below 6 GHz. The work in Reference [11] presented a statistical approach to obtain realistic maximum power levels of 5G gNodeB (gNB) for the assessment of EMF exposure, employing massive MIMO for DL scenarios. An experimental and sequential statistical analysis for the assessment of EMF exposure from a massive MIMO 5G testbed was presented in Reference [12]. The work focused on a 5G DL operating at sub-6 GHz frequency bands in an indoor empty scenario with low topological complexity. The impact of different beam profiles and number of users was assessed by means of a campaign of measurements. Moreover, an EMF exposure assessment from DL base station transmissions in a commercial 5G network was also analyzed in Reference [13]. Finally, the work in Reference [14] presented a comprehensive exposure assessment methodology to measure EMF radiation exposure from DL 5G NR base stations, using conventional spectrum analyzer equipment.

On the other hand, other works in the literature focused on the EMF assessment of the uplink (UL) from the UE in a 5G system. These works usually focused on the exposure assessment of a unique user device and its interactions with the human body. The authors in References [15,16,17,18] presented the impact of EMF exposure from mmWave phased arrays in mobile devices for 5G communication systems. The work presented in Reference [19] investigated the maximum Effective Isotropic Radiated Power (EIRP) that can be achieved at 28 and 39 GHz considering beamforming UE under the constraints of the incident power density regulation limits. Colombi et al. presented in Reference [20] the analysis of radio frequency (RF) energy absorption by biological tissues from mmWave 5G wireless devices in near-field conditions, showing low radiation effects of the near-field body interactions when evaluating EMF compliance at mmWave frequencies. In addition, the authors in Reference [21] presented actual 5G UE output power levels operating in the current commercial 5G networks below 6 GHz, showing that the time-averaged output power levels were, in all cases, well below the regulation limits. Table 1 summarizes the different proposed methodologies for the RF-EMF exposure assessment of 5G communication systems presented in recent years. As it can be seen from the table, there were few works in the literature that presented an approximation in which simulation estimations were simultaneously combined with measurement results. References [6,7,8,9,10,11] presented different methodologies based only on simulation techniques, all for DL and sub-6 GHz frequency bands. Conversely, the works in References [12,13,14] presented EMF exposure assessment methodologies based on measurements campaigns, again at sub-6 GHz frequency bands and DL assessments. Nevertheless, less attention has been given in the literature to the assessment of human EMF exposure considering both DL and UL in complex heterogeneous real-world crowded environments, where multiple wireless communication systems coexist, which is the focus of this work, providing a multi-Gb/s low-latency channel capacity and enhanced mobile broadband experiences to the users.

Accordingly, based on an in-house implemented deterministic 3D ray launching (3D-RL) approach, a novel enhanced simulation tool for RF-EMF exposure assessment is presented, allowing the EMF characterization of complex context-aware scenarios with high node density heterogeneous networks. In this sense, combined wireless communication scenario setups with both DL and UL connections in crowded environments can be analyzed considering realistic operation conditions. The previous version of the simulation tool has already been validated for the assessment of EMF exposure of the current wireless cellular technologies in complex environments [22,23,24]. In Reference [22], the spatial characterization of UL personal RF-EMF exposure in public transportation buses was presented, where worst-case studies considering different user densities and distributions for sub-6 GHz cellular communication systems were evaluated in terms of legislation compliance. The work in Reference [23] reported a simulated and experimental comprehensive analysis of the UL from a 2G–5G cellular system exposure assessment within a public tramway. Although both scenarios, the bus and the tram wagon car, could be considered as complex indoor scenarios in terms of radio wave propagation, the metal structure influence of the tram, as well as the supplying lines and towers and, specifically, its presence in the city central urban districts with huge passenger affluence, involved much more challenging propagation phenomena and, thus, presented higher exposure average levels. Reference [24] presented an environmental RF-EMF radiation exposure assessment from an empirical and simulation approach in public shopping malls, focusing on the current wireless communication systems at sub-6 GHz frequency bands. The main differences of these works, as well as the differences with the presented work, are shown in Table 1.

**Table 1:** Different methodologies for the assessment of RF-EMF exposure for 5G.

Ref.	EMF Assessment		Carrier Frequency		Beamforming		Environment	Simulation	Measurements	Channel Model Analysis	Description
	DL	UL	Sub-6 GHz	mmWave	Fixed Beams	Flexible Beams					
[6]	✓	✗	✓	✗	✗	✓	UMi	✓	✗	3GPP UMi-Street Canyon Model Release 16 [25]	Localization-enhanced pencil beamforming technique, in which the traffic beams are tuned in accordance with the uncertainty localization levels of User Equipment (UE).
[7]	✓	✗	✓	✗	✓	✓	UMa	✓	✗	RT	Computational method to estimate human EMF exposure in DL 5G base stations in outdoor macrocells environments.
[8]	✓	✗	✓	✗	✓	✗	Industrial env.	✓	✗	Hybrid approach: RT/FDTD	Numerical approach for massive MIMO human exposure assessment in industrial environments.
[9]	✓	✗	✓	✗	✓	✓	UMi	✓	✗	Hybrid approach: RT/FDTD	Numerical approach that estimates EMF exposure for 5G massive MIMO considering the effects of electromagnetic coupling between a user and the receiving device.
[10]	✓	✗	✓	✗	✗	✓	UMa	✓	✗	Hybrid approach: RT/FDTD/Network planning methods	Novel method to design massive MIMO 5G networks under power consumption and EMF constraints.
[11]	✓	✗	✓	✓	✓	✗	LoS conditions	✓	✗	-	Model for time-averaged realistic maximum power levels gNBs based on a statistical approach.
[12]	✓	✗	✓	✗	✗	✓	Indoor empty room	✗	✓	-	Statistical assessment from experimental measurements in DL 5G at sub-6 GHz frequency bands considering an empty room with low topological complexity.
[13]	✓	✗	✓	✗	✗	✓	Dense urban area	✗	✓	-	EMF exposure assessment based on real network data from base stations in a commercial 5G network.
[14]	✓	✗	✓	✗	✓	✓	Urban	✗	✓	-	Exposure assessment methodology for measure with common spectrum analyzer equipment 5G NR base stations DL exposure.
[21]	✗	✓	✓	✗	✗	✓	Dense urban area/Urban area	✗	✓	-	EMF exposure assessment based on real network data from 5G UE operating in commercial 5G networks.
[23]	✗	✓	✓	✓	✓	✗	Indoor vehicle	✓	✓	RL [26]	Deterministic model to assess RF-EMF exposure of different systems within indoor metallic vehicles with different users’ densities and distributions, and comparison with current cellular technologies.
[24]	✓	✗	✓	✗	✓	✗	Shopping malls case study	✓	✓	RL [26]	Empirical and deterministic model to assess RF-EMF exposure on sub-6 GHz shopping malls case study.
This work	✓	✓	✗	✓	✓	✓	Indoor complex env.	✓	✓	RL [26]	Empirical and deterministic model to assess RF-EMF exposure on mmWave high-node density complex heterogeneous environments, with high topological complexity where all the scatterers are included.

UMi: urban microenvironment; UMa: urban macroenvironment; RT: ray tracing; RL: ray launching; FDTD: Finite Difference Time Domain.

In this work, a step further is proposed for the EMF exposure assessment technique by considering mmWave frequency bands, allowing beamforming emulation with flexible beams, single-user MIMO (SU-MIMO) and multi-user MIMO (MU-MIMO) for both DL/UL (although, nowadays, MU-MIMO is only a DL feature, the simulation tool can consider MU-MIMO also in UL to emulate a potential technology development taking advantage of cooperative receiver schemes), different heterogeneous wireless communication technology analyses, complex indoor environments considering all the scatterers of the scenario, UE and gNB scenario densification and multifrequency operation, etc. The enhanced 3D-RL simulation technique has been implemented for the EMF exposure assessment of a potential complex indoor crowded scenario where two wireless communication systems will coexist in the mmWave frequency range. The selected scenario is a two-floor partial area of a new library building located on a university campus in which two different systems have been implemented: 5G personal mobile communications at frequency range 2 (5G-FR2) and wireless data access services WLAN 802.11ay at 60 GHz. The novel aspects of this work in relation with the previous works are the following:

- High-node user density environments: Software implementations have been performed in the 3D-RL algorithm kernel to allow user densification scenarios. By means of multiple integrated simulations considering high-node user density scenarios of increased complexity, the raw data is merged and collected in a new module to provide accurate final results. Following this procedure, the enhanced simulation tool is able to adequately reproduce the behavior and influence of environmental RF-EMF radiation exposure, considering different approaches: from a specific local exposure assessment in a particular communication beam to the overall exposure distribution in all the selected scenarios.
- Beamforming techniques: The consideration of 5G MIMO antennas and beamforming is provided by means of a new enhanced beamforming strategy that has been implemented using postprocess modules and multiple integrated simulations in order to simulate real antenna operations under future expected severe UL and DL conditions in complex high-node user dense context-aware heterogeneous environments.
- Complex heterogeneous environments at mmWaves: The consideration of heterogeneous environments in terms of electromagnetic fields and, specifically, in the exposure assessment and dosimetric characterization, is pivotal due the advent of new wireless communication systems and their unstoppable widespread use. It is a reality that current and future communication systems have become and will be increasingly heterogeneous, providing services and applications relying on coexisting merged heterogeneous networks in order to comply with coverage/capacity relations. Technically, when considering a complex heterogeneous system in the proposed enhanced simulation tool, a full analysis of the spectrum use needs to be performed in order to provide an adequate exposure assessment and evaluation based on the fact that multiple systems are operating at the same time in different frequency ranges, increasing the overall spectral usage. Thus, this corresponding electromagnetic influence is characterized in a new synchronized and integrated module by implementing merging techniques, as more intensive spectrum uses with multiple systems have unique characteristics. In addition, these challenging environments need to consider high complex scenario spatial designs in terms of the morphology and topology, with special attention on the scatterers’ frequency dispersive material properties. In this sense, the material properties’ database library of the new enhanced version of the simulation tool has been upgraded to consider material properties up to mmWaves.

The rest of the paper is organized as follows. Section 2 presents the Materials and Methods, where the enhanced EMF exposure tool is presented, as well as the scenario description with the considered simulated study cases and the measurement campaign in order to validate the proposed EMF-enhanced technique. Then, Section 3 reports the simulation results with the analysis of the different study cases and the measurement results. Finally, the conclusions are presented in Section 4.

## 2. Materials and Methods

### 2.1. Ray Launching Technique

**Figure 1:**
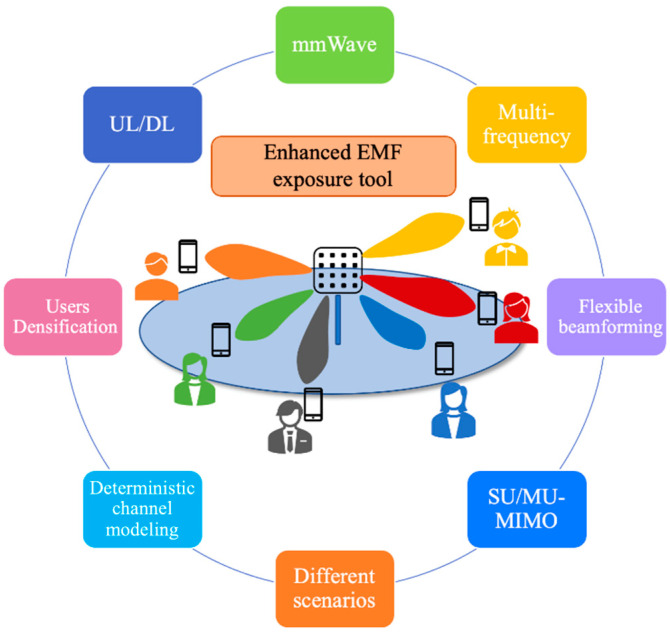
Schematic view of the distinctive characteristics of the enhanced EMF exposure simulation tool.

As it has been previously introduced, the EMF exposure simulation tool has already been used in previous works in order to analyze nonionizing radiation exposure in complex indoor environments when multiple cellular technologies coexist. In this work, a step further is proposed in order to analyze an EMF exposure assessment in a crowded indoor complex environment where 5G-FR2 and WLAN 802.11ay wireless communication systems operating at 28 and 60 GHz, respectively, coexist. For that purpose, the EMF exposure tool has been enhanced in order to consider the distinctive characteristics of these wireless communication systems. The most significant ones are presented in Figure 1 and can be described as follows: (i) possibility to simulate mid-band frequencies and mmWave system operations, (ii) multifrequency operations, (iii) flexible beamforming depending of the number of UEs served, (iv) possibility of considering SU/MU-MIMO in both UL and DL, (v) consideration of different complex scenarios with all the scatterers/obstacles within them, (vi) a geometric-based deterministic channel model (GBDM) based on an in-house three-dimensional ray launching (3D-RL) technique, (vii) feasibility of the densification of the UE and base stations/access points and (viii) UL and DL EMF exposure can be assessed in a high-user density indoor/outdoor environment.

GBDM based on full-wave techniques are considered highly accurate, as they take into account all the geometry and obstacles of the environment, but they present the disadvantage of a higher computational cost, which can be unaffordable for typical real-world scenarios [27,28]. In this sense, GBDM based on ray tracing or ray launching techniques such as the in-house 3D-RL algorithm presented in this work achieve good accuracy with a reasonable computational load for typical real-world scenarios [29,30]. Traditionally, ray launching and ray tracing are both classified as ray tracing methods, although, more recently, both methods have been distinguished. The differences are principally due to different methodologies. The principle of ray launching techniques is to launch sets of test rays from the transmitter to determine the true path by looking for the rays that arrive at the receiver. Conversely, in ray tracing methods, the basic principle is to compute the image of the transmitter or the receiver to find the reflected paths by using walls and furniture.

The proposed in-house 3D-RL algorithm is based on Geometrical Optics (GO) and the Uniform Theory of Diffraction (UTD). It has been previously validated in different indoor/outdoor complex environments [31,32] for different applications, such as vehicular communications [33,34], Intelligent Transportation Systems (ITS) [35,36,37], interference analysis [38,39], Wireless Local Area Networks (WLAN) [40,41], Wireless Sensor Networks (WSNs) [42,43] or Public Land Mobile Networks (PLMN) [23,24], among others. These applications have been validated for different operating frequencies (from mid-bands to mmWave frequency bands) and different wireless communication system configurations, achieving, in general, a Root Mean Square Error (RMSE) of 3–6 dB, an absolute mean error of 4–5 dB and a Standard Deviation (SD) of 1–7 dB. The algorithm basis is that a certain number of rays is launched from the transmitter to the receiver with a determined angular and spatial resolution. Depending on the UE and access point locations in the considered scenario, the angles for the different beams are defined and then launched from the transmitter (which can be one or more UE or access points), depending on the selected input parameters of the algorithm. The implemented flexible beamforming in the enhanced simulation tool consists of the simulation of a set of spatial orientations (beams) with a defined angular and spatial resolution in the 3D scenario. The algorithm beamforming strategy is based on the instantaneous mapping of each defined beam in the considered scenario. At this step, all possible cases of angular variation can be considered by simulation (both in the base station and in the UE). Then, once all simulations are performed, in a postprocessing algorithm step, flexible beamforming takes place. By considering all the simulation-based cases, the beamforming can be defined and analyzed depending on the wireless system operating conditions under analysis.

It is worth noting that the algorithm is not influenced by the type of beamforming strategy considered for the wireless system, exhibiting, in principle, no limitation in this sense. This is given by the fact that, considering the selected beamforming strategy, the corresponding sequence of simulations to be performed is scheduled. Once these simulations have been performed, in the simulation postprocessing step, the different 3D spatial planes can be overlapped, obtaining the final results.

The algorithm principle is the following: when a ray hits an obstacle, reflected and refracted rays are created, and when a ray hits an edge, a new family of diffracted rays is created. These new rays are created according to the material properties of all the obstacles within the environment for the operation frequency of the system under analysis.

The electric field *E* created by GO and the diffracted electric field created by UTD are calculated by [44]

(1) $$E_{\mathrm{GO}}=\sqrt{\frac{P_{\mathrm{rad}}D_{t}\left(\theta_{t},\;\emptyset_{t}\right)\eta_{0}}{2\Pi }}\;\frac{e^{-j\beta_{0}r}}{r}{\;Χ}^{⊥∥}L^{⊥∥}$$

(2) $$E_{\mathrm{UTD}}=e_{0}\frac{e^{-jks_{1}}}{s_{1}}D^{s,h}\sqrt{\frac{s_{1}}{s_{2}\left(s_{1}+s_{2}\right)}}e^{-jks_{2}}$$

where $\beta_{0}=2\pi f_{c}\sqrt{\varepsilon_{0}\mu_{0}}$, $\varepsilon_{0}$= 8.854 × 10^−12^ F/m, $\mu_{0}$ = 4*π* × 10^−7^ H/m and $\eta_{0}\;$= 120 *π* Ω. *P*_rad_ is the radiated power of the transmitter antenna. $D_{t}\left(\theta_{t},\;\emptyset_{t}\right)$ is the directivity of the beam radiation pattern where rays are launched, as defined in the spherical coordinate system at an elevation angle $\theta_{t}$ and an azimuth angle $\emptyset_{t}$. For each polarization, ${Χ}^{⊥∥}$ and $L^{⊥∥}\;$ are the polarization ratio and path loss coefficients, *r* is the distance in the free space and *f_c_* is the transmission frequency. In the diffracted electric field created by UTD, $D^{s,h}\;$ are the diffraction coefficients for soft and hard polarization determined by considering the edge-fixed incidence plane, $e_{0}\;$ is the free-space field strength, *k* is the propagation constant and $s_{1}$ and $s_{2}$ are the distances from the source to the edge and from the edge to the receiver point [44,45]. The diffraction phenomena can be activated or disactivated by the user, as one of the simulation software parameters. The complete scenario is divided in a predetermined 3D mesh, and the total electric field is calculated with the sum of the reflected, refracted, diffracted and incident electric vector fields at an instantaneous time inside each cuboid of the defined mesh. From these results, the incident power density can be calculated as the modulus of the complex Poynting vector [46]:

(3) $$S_{inc}=\left|E\times H^{*}\right|$$

where E is the E-field in volts per m (V/m), and $H^{*}$ is the complex conjugate of the magnetic field in amperes per m (A/m). In the case of the far field, the incident power density is derived as

(4) $$S_{inc}=\frac{\left|E^{2}\right|}{Z_{0}}=Z_{0}\left|H^{2}\right|$$

where $Z_{0}$ is the characteristic impedance of free space (i.e., 120 π Ω). Some restrictions apply when considering RL simulation techniques concerning near field possible discrepancies and uncertainties in the proximity of the transmitter location. The presented environmental RF-EMF assessment study considers only far field conditions and the influence of the user presence, density and distribution but does not include an in-body assessment, as RT techniques are not suitable for those purposes. Consequently, in order to prevent unreliable near field results, an adaptable/dynamic exclusion area of 5λ (λ as the wavelength of the propagating wave under consideration) is applied around the transmitter antenna, considering the different frequencies under analysis [44].

**Figure 2:**
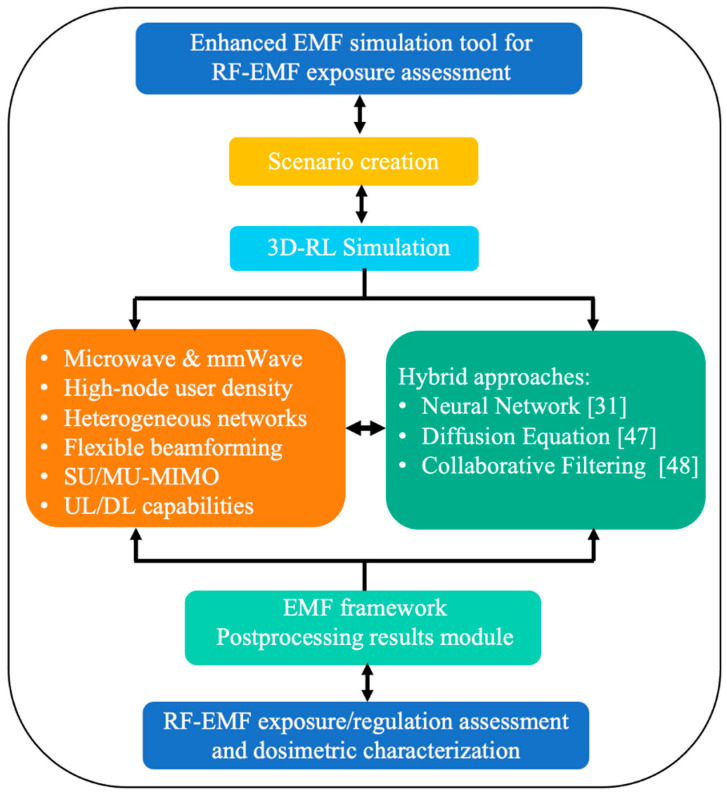
Flowchart of the enhanced RF-EMF exposure and regulation assessment simulation tool.

Furthermore, the in-house developed 3D-RL tool allows the possibility of applying hybrid techniques to decrease the computational cost given by the complexity of the environment under analysis. These hybrid techniques are the Neural Network (NN) module [31], the Diffusion Equation module [47] or the Collaborative Filtering module [48], which achieve accurate simulation results while the computational load decreases considerably. Figure 2 presents a flowchart of the enhanced 3D-RL simulation methodology for RF-EMF radiation exposure and regulation assessment, where the main steps of the algorithm, along with its main capabilities, are presented.

### 2.2. Scenario Description

**Figure 3:**
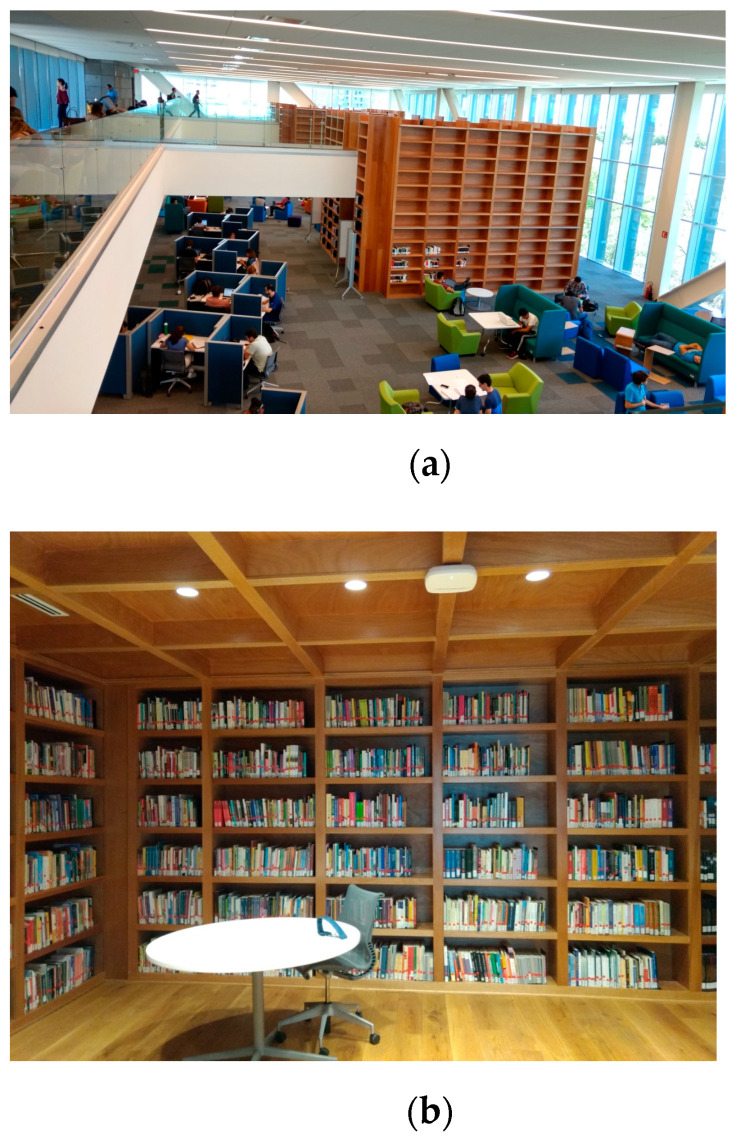
Considered real scenario to analyze EMF exposure with high-user density for future wireless communications systems: (**a**) aerial view of the complete scenario with two floors and (**b**) first floor inside the wooden bookshelves.

**Figure 4:**
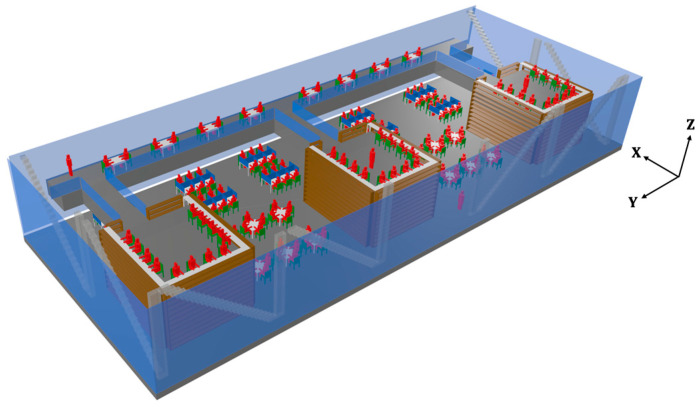
Rendered view of the simulated scenario by means of the 3D-RL technique.

The selected scenario is a complex indoor environment that corresponds with two floors of a new library building located on the university campus of Tecnologico de Monterrey, Campus Monterrey in Mexico. Figure 3a shows a picture of the real scenario. The first floor has workspaces in the open area, as well as within the wooden bookshelves, as can be seen in the detailed picture of Figure 3b, which represents the inner part inside the wooden shelf. The second floor has an aisle with workspaces in the left part of the scenario and three aisles to access the second floor of workspaces within the wooden bookshelves. Figure 4 shows the rendered 3D view of the simulated scenario, where all the objects within the environment have been considered.

In the considered scenario, 9 Wi-Fi access points are currently installed, 6 of them located on the second-floor ceiling at a 6-m height and 3 more are placed within the workspaces inside the wooden bookshelves. These access points can be seen in the central part of the ceiling shown in Figure 3.

**Figure 5:**
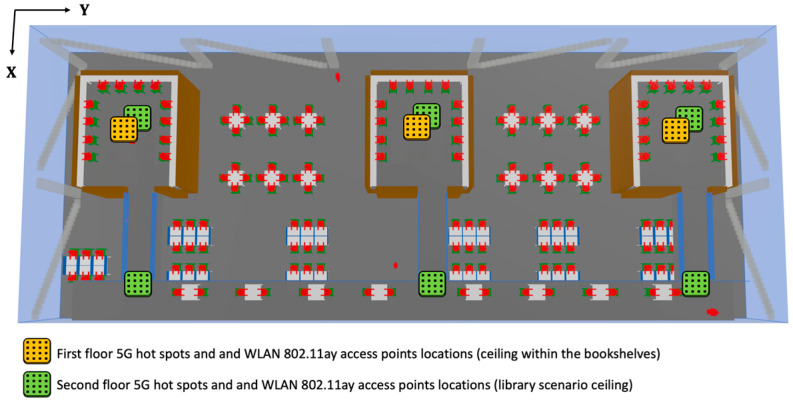
The 5G hot spots and WLAN 802.11ay access points locations co-sited with the current Wi-Fi access points in the scenario under consideration.

From an economical and aesthetic point of view, it is desirable to reuse existing indoor access point locations in the library as new communication systems are deployed in order to provide greater capacity, higher data rates and lower energy consumption in future networks. Thus, in the proposed future scenario, we have considered that the existing current Wi-Fi access points will be mounted co-sited with 9 mmWave gNodeB (gNB) hot spots operating at 5G-FR2 and 9 WLAN 802.11ay access points operating at 60 GHz. Figure 5 presents a schematic overview of the locations of all these proposed access points.

The total area to be assessed by the future deployment is approximately 640 m^2^ for the first floor, plus 60 m^2^ that corresponds with the corridors and floor in the upper part of the bookshelves on the second floor, which is a total of 700 m^2^ of floor space available for users. Likewise, the maximum seated workspaces in the selected area of the campus library are 202; out of which, 52 are on the second floor and 150 on the first floor. Thus, the maximum high-density occupation considered in the simulations is that all workspaces are occupied with an active user (i.e., a user making use of his/her full network capacity). Then, as the medium-density occupation, 70% of the high density is considered, which corresponds with approximately 140 active users.

For the simulations, all the dispersive material properties of all the objects within the environment are taken into account for the frequency of the operation under analysis (considering the conductivity and relative dielectric permittivity). These properties are presented in Table 2 [26]. Specifically, the skin tissue model is considered for tissues of high-water contents as a function of the wavelength in the air [49].

Table 3 presents the main input parameters for the 5G and Wi-Fi 802.11ay simulation setup in the scenario under analysis. We use 64 element antennas for the access points, which is a typical value for an indoor environment at mmWave frequency bands [50], and 8 antennas per polarization for the UE antenna. For comparison purposes, a uniform power distribution per user in the scenario is considered, where each beam always transmits at the same power in the DL direction (15 dBm) and in the UL direction (10 dBm), i.e., no traffic adaptation mechanisms are applied to the power radiated by the beam.

As previously stated, future wireless communication systems such as 5G-NR or WLAN 802.11ay will have benefits in several domains, such as the time domain with variable TDD schemes. In this sense, in TDD, the UL is separated from the DL by the allocation of different time slots within the same frequency band.

From an exposure assessment point of view, the realistic maximum exposure should be proportional to the fraction of the DL transmission time to the total time given by [11]

(5) $$F_{\mathrm{TDD}}=\frac{\mathrm{DL}\;:\mathrm{UL}}{\mathrm{DL}\;:\mathrm{UL}+1}$$

where DL:UL denotes the DL/UL transmission configuration (ratio of DL transmission time to UL transmission time). In this paper, *F*_TDD_ = 0.75 is assumed as a reasonable value for future wireless communication systems [11].

In order to analyze the EMF exposure in the considered scenario, three different cases are considered for comparison purposes. These three cases are the following:

- Case I: We consider that 100% of the users in the library are connected to 5G and no one to WLAN 802.11ay, so we can assess EMF exposure only from the 5G communication system at 5G-FR2 (@28 GHz) in a crowded environment.
- Case II: We consider that 100% of the users in the library are connected to WLAN 802.11ay and no one to 5G, so we can assess EMF exposure only from the WLAN 802.11ay (@60 GHz) communication system in a crowded environment.
- Case III: We consider a case where both systems coexist, and 30% of the users are connected to 5G and 70% to WLAN 802.11ay. The higher percentage for the WLAN versus 5G is considered, because in an indoor environment where both systems will be deployed, as in a campus library, we assume that students will prefer to use WLAN for free instead of their own cellular data.

For all cases, among the users of WLAN 802.11ay and 5G, we consider that 70% are using DL and 30% UL. For reference, the exact distribution of active users for the different cases is presented in Table 4.

### 2.3. Measurement Campaign

In order to validate the proposed EMF exposure-enhanced algorithm, a campaign of measurements is performed within the real scenario of the new building library presented in Figure 3 for both the 28 and 60 GHz operating frequencies. For that purpose, two different measurement setups are configured for the 28 and 60 GHz frequency bands. Table 5 presents the different setups, along with the used equipment and its description.

**Figure 6:**
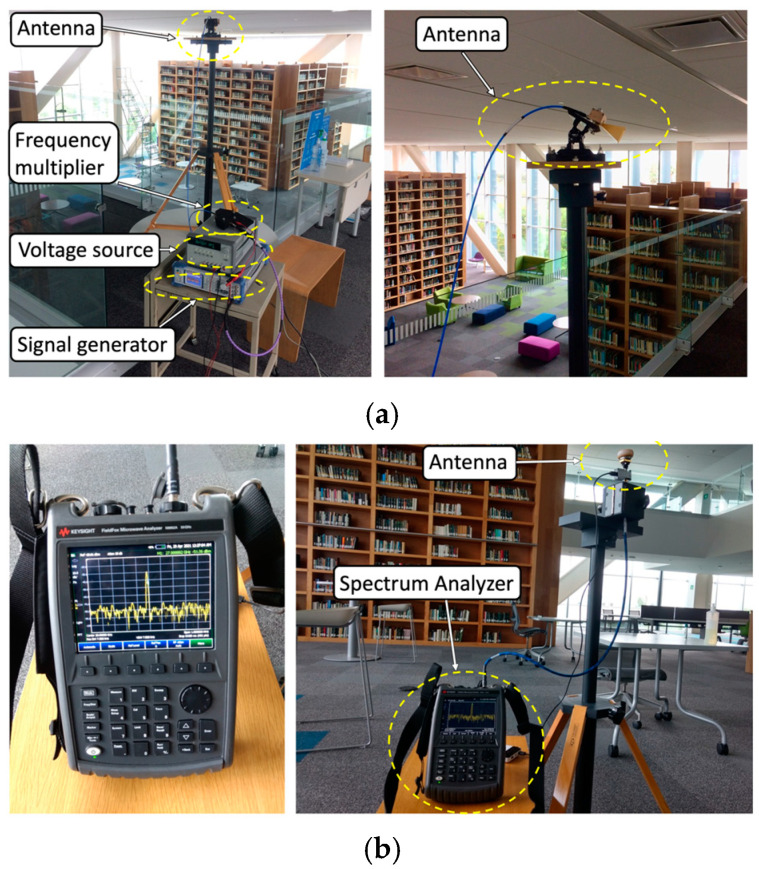
Measurement setup at 28 GHz frequency on the first and second floors of the library: (**a**) transmitter and (**b**) receiver.

**Figure 7:**
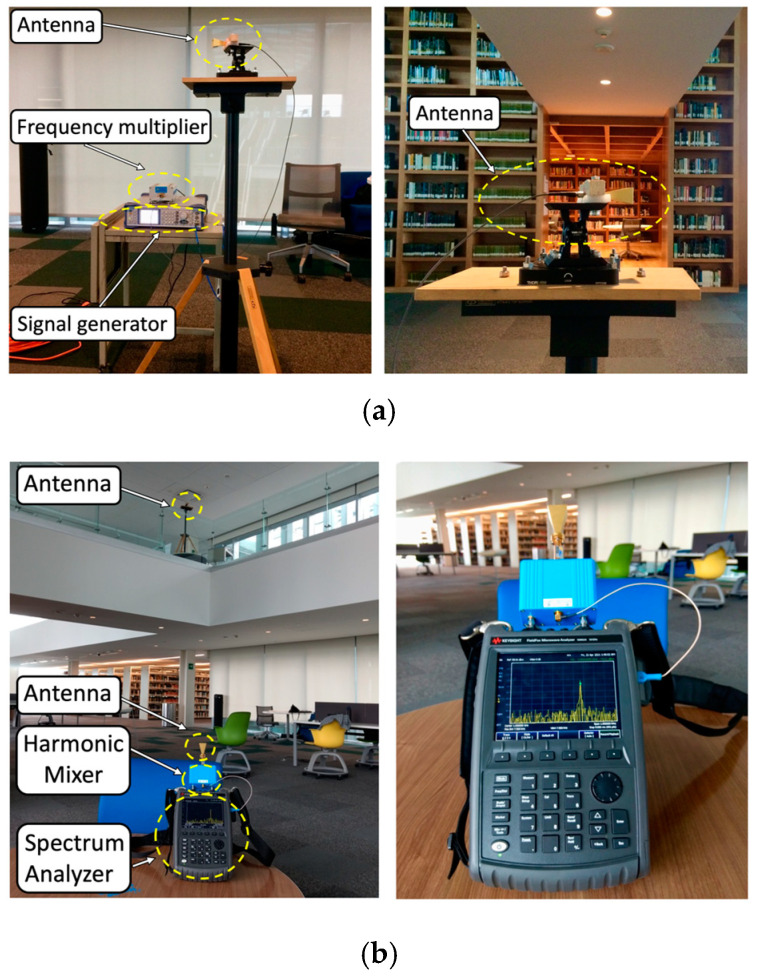
Measurement setup at 60 GHz frequency on the first and second floors of the library: (**a**) transmitter and (**b**) receiver.

Figure 6 and Figure 7 show examples of the transmitter and receiver setups in the experimental campaign of measurements for the 28 and 60 GHz operating frequencies, respectively.

**Figure 8:**
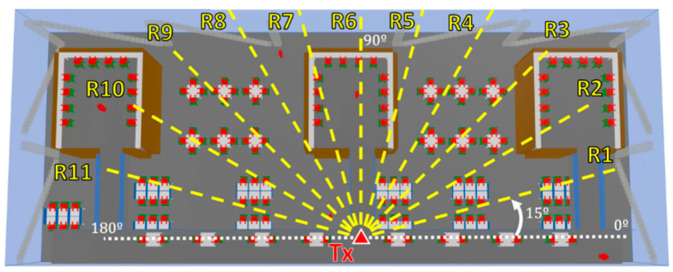
Transmitter antenna beamforming and radials of the receiver measurement points for the 28 and 60 GHz operating frequencies when the transmitter is placed on the first floor.

For each operating frequency, two different measurement setups are considered, the first one with the transmitter placed at 1.3 m on the first floor and the second one with the transmitter placed at 1.85 m on the second floor. The antennas are placed with the aid of a tripod constructed from wood and nylon materials (AT-812 Antenna Tripod from Com-Power Corporation (Silverado, CA, USA) (see Figure 6 and Figure 7 for reference). When considering the first setup case (transmitter antenna on the first floor), an antenna angular sweep from 0 to 180° with steps of 15° is performed, while measurements points are taken for each antenna position along the radial distance each 2 m at the same height as the transmitter. The minimum and maximum radial distances between the transmitter and receiver antenna are 22 m and 11 m, respectively. The transmitter antenna is directed towards the receiver points with the aid of an antenna positioner from Thors Labs (Newton, NJ, USA). The antenna alignment is performed with a laser pointer. By means of this setup of 15° radials, with a measurement distance of 2 m per measurement location point, the complete library area of the first floor is characterized, allowing simulation comparisons. For clarification purposes, the different measured radials for each transmitter antenna beamforming are depicted in Figure 8.

**Figure 9:**
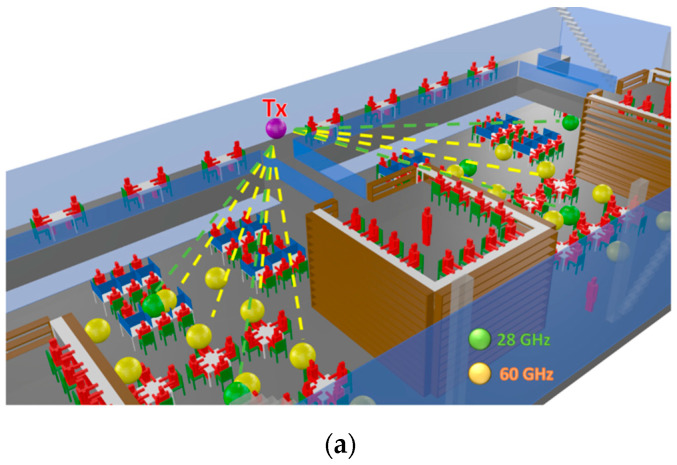

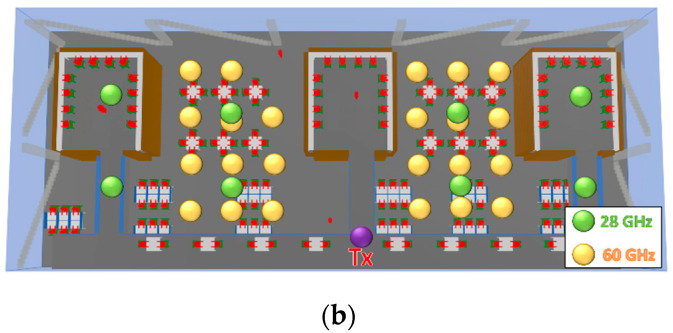
Transmitter antenna beamforming and receiver measurement points for the 28 and 60 GHz operating frequencies when the transmitter is placed on the second floor: (**a**) 3D view and (**b**) 2D view.

For the second setup case (transmitter antenna placed on the second floor), the antenna is aligned with different fixed receiver points on the first floor with the aid of the antenna positioner and the laser pointer in order to perform the different measurements for each operating frequency. Figure 9 shows the considered transmitter antenna position on the second floor and the different measured receiver points for both frequencies.

## 3. Results and Discussion

### 3.1. Simulation Results

**Figure 10:**
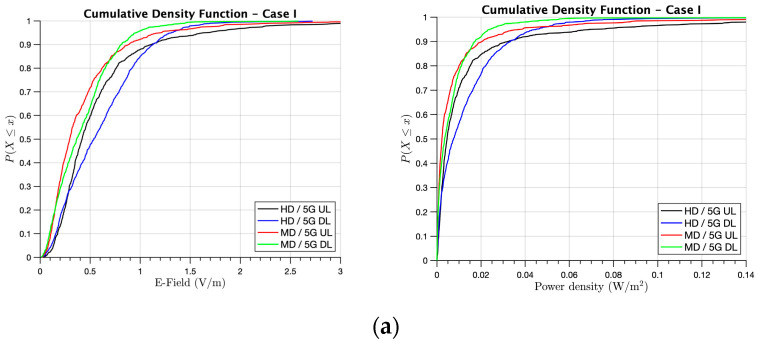

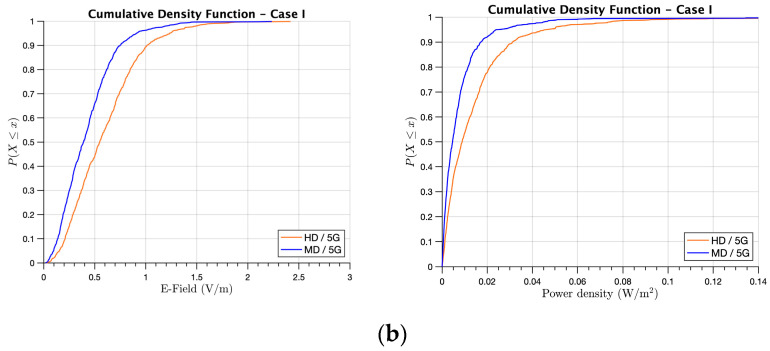
CDF of the received E-field and power density for Case I at the XY bi-dimensional plane at head height when the people were working on the first floor of the library: (**a**) UL and DL comparisons for HD and MD and (**b**) the total E-field exposure and power density for HD and MD.

**Figure 11:**
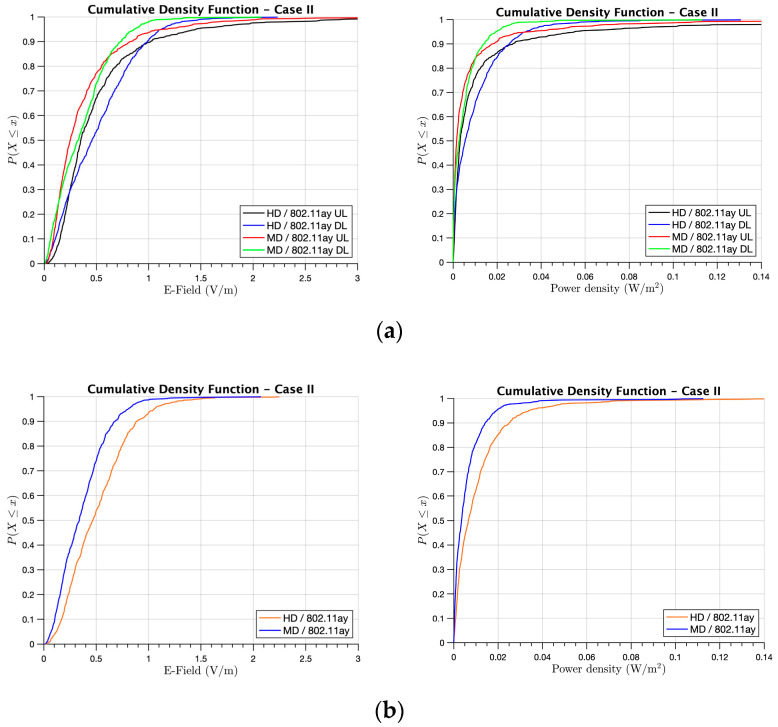
CDF of the received E-field and Power density for Case II at the XY bi-dimensional plane at head height when the people were working on the first floor of the library: (**a**) UL and DL comparison for HD and MD and (**b**) the total E-field exposure and power density for HD and MD.

**Figure 12:**
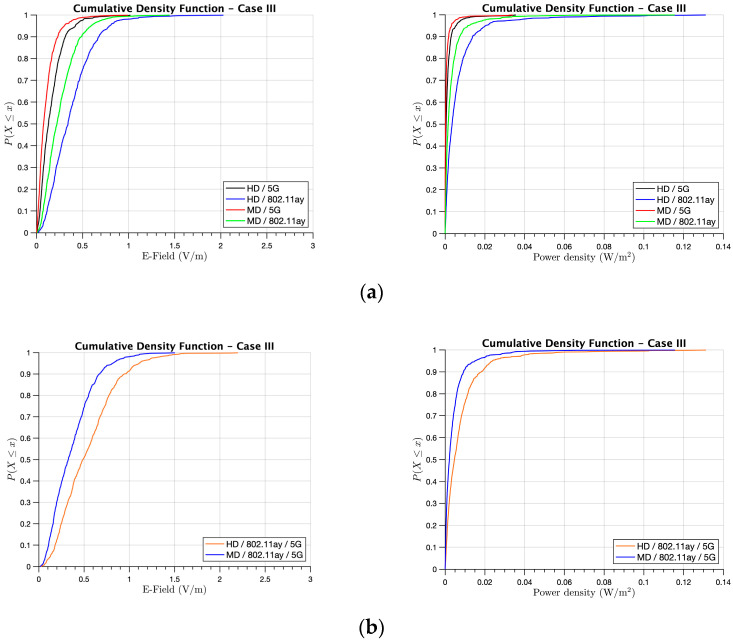
CDF of the received E-field and power density for Case III at the XY bi-dimensional plane at head height when the people were working on the first floor of the library: (**a**) 5G and 802.11ay comparison for HD and MD and (**b**) the total E-field exposure and power density for HD and MD.

From all the 3D-RL simulation results, the relevant E-field and power density characterization, impact and behavior patterns were obtained:

Firstly, Figure 10, Figure 11 and Figure 12 present the Cumulative Density Function (CDF) for the received E-field and power density values in V/m and W/m^2^, respectively, at the XY bi-dimensional plane of a 1.6-m height, which corresponded with the head height of the seated active users at the first floor of the library. First, Figure 10 presents the results for Case I, in which we considered that 100% of the users in the library were connected to 5G and no one to WLAN 802.11ay. In Figure 10a, an assessment between the 5G UL and DL connections was done, with different user densities (i.e., MD and HD). It could be seen that the, in the higher user density scenario, both UL and DL had higher E-field and power density values than the MD scenario in the order of 0.1–0.2 V/m or 0.005–0.01 W/m^2^, approximately. In addition, it was observed that the DL connection results presented, on average, more E-field levels than UL for this case. This was due to the fact that we considered 70% of active users as using DL and 30% UL. Although the analyzed height was closer to the active user in the UL, the influence of the E-field/power density levels by nearby users due to UL was lower in average than the DL from the access points in the order of 0.1 V/m or 0.005 W/m^2^. In order to have insight into the differences of both UL and DL in the different user densities considered, Figure 10b shows the CDF for the total E-field and power density values received at the same height in the scenario. As stated before, in order to calculate the realistic maximum exposure, *F*_TDD_ was applied to calculate these results considering the ratio of the DL transmission time to UL transmission time. It could be seen that the total E-field exposure was higher for HD yet lower than 2 V/m for all user locations in the considered scenario. The same trend could be seen for the power density values, obtaining higher values for HD yet lower than 0.1 W/m^2^ for all cases.

Secondly, Figure 11 presents the results for Case II, in which we considered that 100% of the users in the library were connected to WLAN 802.11ay and none to 5G. Following the same E-field and power density pattern than in the previous case, it could be seen that higher E-field and power density levels were obtained for the HD case yet remained for all cases below 2 V/m and 0.1 W/m^2^, respectively. In comparison with Case I, the increasing frequency up to 60 GHz resulted in slightly lower exposure levels (in the order of 0.1–0.2 V/m for the E-field and 0.005–0.01 W/m^2^ for the power density) than in a 28 GHz 5G system, as can be observed in Figure 11b, where the maximum exposure was calculated for Case II. This phenomenon was the result of the higher signal attenuation at higher frequencies.

Finally, Figure 12 presents the most realistic case, where both systems coexisted (i.e., 30% of the users were connected to 5G and 70% to WLAN 802.11ay). In Figure 12a, the difference of the maximum exposure values considering the ratio of DL transmission time to UL transmission time for the different systems and user densities is shown. From the obtained E-field and power density results, the maximum exposure values corresponded to the HD 802.11ay system, which was the predominant system in this case (realistic case), yet remained with very low levels: E-field levels lower than 2 V/m and power density levels lower than 0.1 W/m^2^ for all cases. In order to gain insight into the maximum exposure levels that potential users in this scenario could be exposed to, Figure 12b presents the total E-field and power density levels received for both systems operating at the same time for the HD and MD cases. In conclusion, for the worst case of HD when all working places of the library had active users using one of the two considered systems, the maximum E-field and power density exposure levels remained below 2 V/m and 0.1 W/m^2^, respectively, for all user locations.

**Figure 13:**
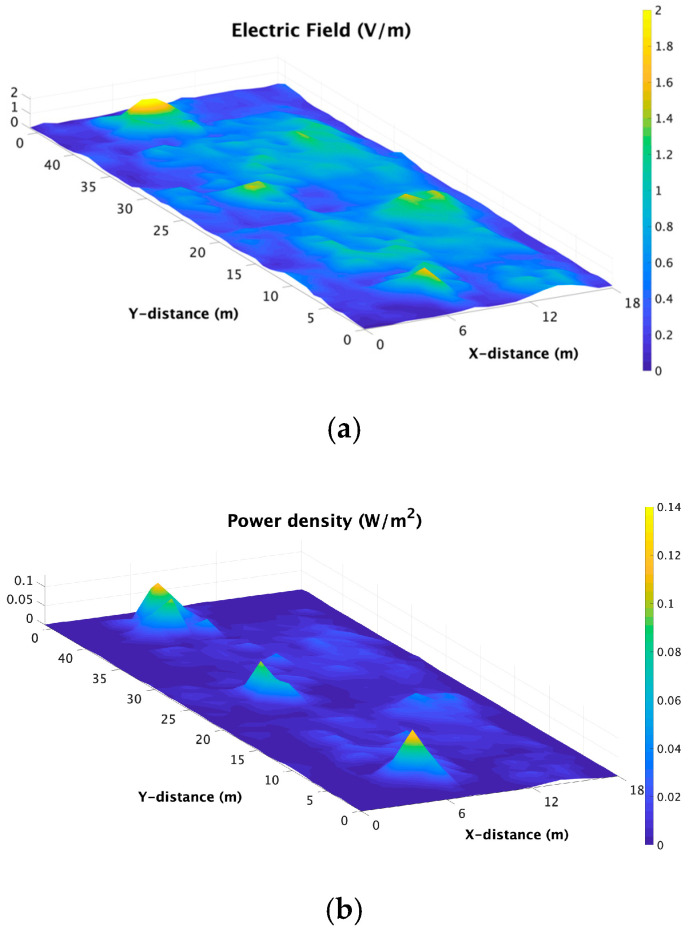
E-field and Power density distribution levels of the XY bi-dimensional plane at the working spaces at head height of seated people on the first floor of the library for Case III HD: (**a**) E-field and (**b**) power density.

In addition, Figure 13 shows the environmental E-field and power density exposure levels considering the XY bi-dimensional plane at the height of a seated person in a working space on the first floor of the library for Case III HD (realistic case conditions). From the results obtained, we could conclude that there were no significant variations in the E-field or power density level distribution, and the highest levels were encountered within the user working spaces inside the bookshelves, as these spaces were subject to higher reflection phenomena produced by the bookshelf’s walls. Nevertheless, as it has been clearly remarked before, the higher E-field values encountered remained lower than 2 V/m, and the higher power density levels remained lower than 0.1 W/m^2^. The current regulation limits based on the ICNIRP guidelines [54] state that when determining the compliance for frequencies between 2 and 300 GHz, the incident power density must be considered. Thus, it can be stated that the obtained power density for all the cases was far below the current regulation limit for the general population for these frequency bands, which was 10 W/m^2^ [54].

### 3.2. Measurement Results

A complete campaign of the measurements was performed in the library scenario under analysis to validate the enhanced simulation tool for RF-EMF exposure assessment at 28 and 60 GHz following the previously described measurement guidelines in Section 2.3.

**Figure 14:**
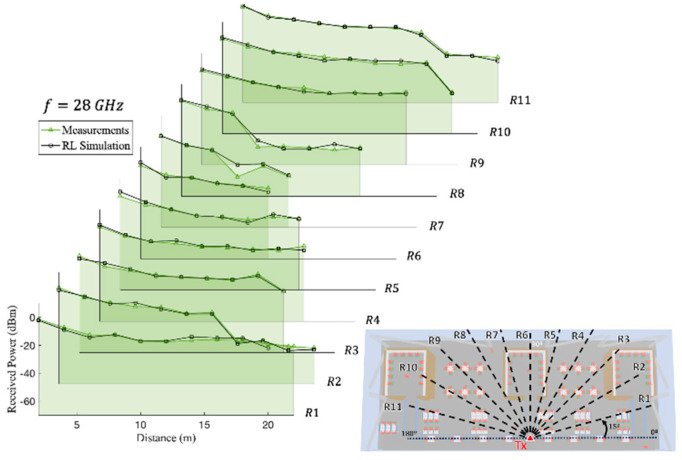
Experimental measurements and 3D-RL simulation comparison at 28 GHz frequency along the different linear radial distributions R_1_, …, R_11_ depicted in Figure 8.

**Figure 15:**
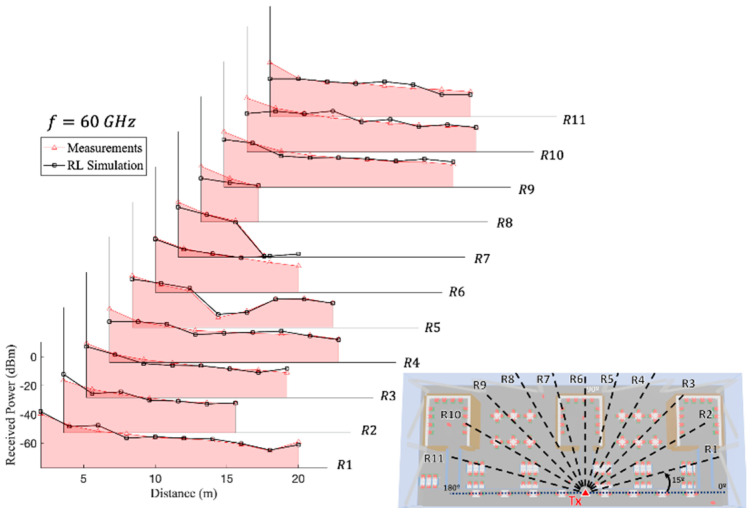
Experimental measurements and 3D-RL simulation comparison at 60 GHz frequency along the different linear radial distributions R_1_, …, R_11_ depicted in Figure 8.

Figure 14 and Figure 15 present the received power comparison between the experimental measurements and the 3D-RL simulation at the 28 and 60 GHz operating frequencies, respectively, along the different radials of the transmitter antenna beamforming (see Figure 8 for reference) when the transmitter was placed on the first floor. In this case, most of the measurements were performed under line-of-sight (LOS) conditions, but the measurements performed within the bookshelves were done under non-line-of-sight (NLOS) conditions (the indoor environment within the bookshelves can be seen in Figure 3). LOS conditions can be considered as a higher risk situation in terms of the nonionizing radiation exposure levels. As it can be seen from the comparisons, the simulation and measurements were in good agreement for both cases. Table 6 shows the differences between the measurements and simulation estimations for both frequencies at each linear distribution radial, ranging from 1.20–3.59 dB for the 28 GHz frequency band to 1.40–5.09 dB for the 60 GHz frequency band. This difference reflects both uncertainties in the measurements and assumptions made in computing. The maximum level of power using the Max Hold function in the handheld spectrum analyzer was used for the measurement results when compared to the simulations. However, we experimentally observed in the campaign of measurements that they fluctuated, with an average value of approximately 2.5 dB.

**Figure 16:**
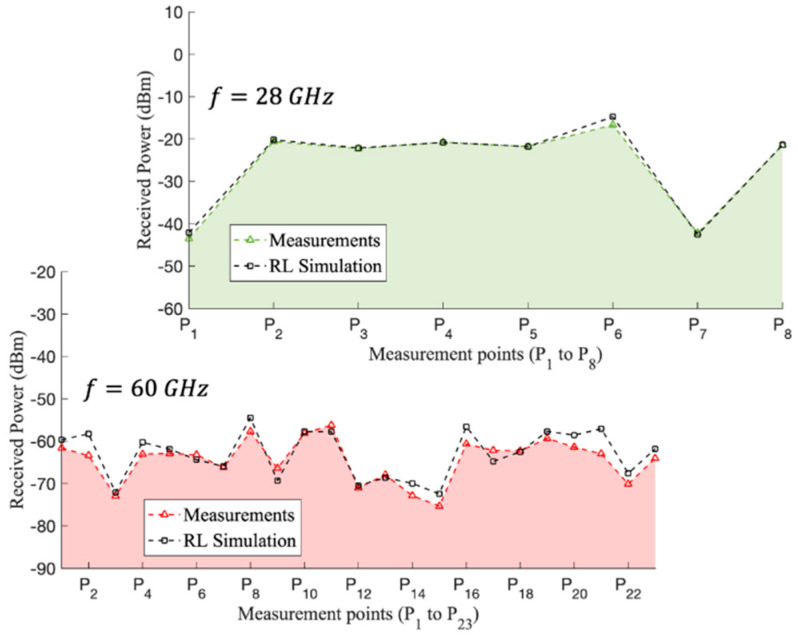
Experimental measurements and 3D-RL simulation comparisons at the 28 and 60 GHz frequencies along the different points depicted in Figure 9.

Moreover, Figure 16 presents the experimental measurements and 3D-RL comparisons for both frequencies (28 and 60 GHz) at the different points represented in Figure 9 when the transmitter was placed on the second floor of the scenario (see Figure 9 for reference). In this case, all measurement points were under LOS conditions. When comparing LOS condition measurement spatial points, it did not mean that we compared only the LOS component, since, although they were under LOS conditions, we also detected multipath components, which could be from different phenomena, such as a reflected, transmitted or diffracted field. The in-house enhanced simulation EMF exposure software considered the full three-dimensional characteristics of the scenario at the geometry level and clutter location, as well as the dispersive properties by means of the conductivity and relative permittivity, of all the obstacles within the environment. In this way, and taking into account that the rays were launched in a volumetric way, the impact of everything that surrounded the radiant sources was considered, whether they were under LOS or NLOS conditions. In addition, the differences between the simulation and measurement results represented in Figure 16 at both frequencies are summarized in Table 6, showing good agreement between them. Finally, the total mean difference is also calculated in Table 6, achieving 1.67 dB at 28 GHz and 2.92 dB at 60 GHz, which validated the potential use of the proposed simulation tool for the RF-EMF exposure assessment before the deployment of 5G and 802.11ay systems at complex indoor environments.

## 4. Conclusions

In this work, environmental RF-EMF exposure was assessed from an empirical and modeling approach in a complex heterogeneous indoor environment when considering dense personal mobile communications operating at 28 and 60 GHz. For that purpose, an enhanced in-house deterministic 3D-RL simulation tool for a RF-EMF exposure assessment was proposed, allowing E-field and incident power density level characterizations in different complex heterogenous scenarios of increased complexity. Realistic operation conditions were considered, as well as different user distributions and densities, emulating advanced mmWave communication systems with directive antennas and MIMO beamforming techniques.

From the obtained results, a discussion regarding the contribution, impact and health effects of the coexistence of multiple heterogeneous networks and services was provided. The main conclusion that must be stated is that wireless communication systems operating at both the 28 and 60 GHz frequency bands considering realistic and worst-case scenarios in terms of user densities, as well as directive antennas and beamforming techniques, generated environmental E-field exposure levels lower than 2 V/m and incident power density levels lower than 0.1 W/m^2^ for all the analyzed cases. Therefore, the compliance with the current established international regulation limits with the exposure levels for the general population far below the aforementioned limits (10 W/m^2^ [54]) was verified even in the worst-case conditions and, at the same time, denying the hypothesis of an increase in the total RF-EMF exposure by the use of mmWave frequencies or directive antennas in complex dense heterogeneous indoor environments. In this sense, these results guaranteed that E-field distributions and received power levels within the complete scenario under analysis were below the thresholds and, hence, complied with the current regulatory frameworks in relation with the health assessment.

Moreover, the proposed simulation methodology was validated with a complete empirical campaign of measurements, showing good agreement with the experimental results. Thus, the obtained measurement datasets and simulation estimations, along with the presented enhanced 3D-RL simulation tool, could be a reference approach for the design, deployment and exposure assessment of the current and future wireless communication systems, where complex context aware scenarios with massive high-node density heterogenous networks are expected in the mmWave frequency range.

## Figures and Tables

**Table 2:** Material properties for the 3D ray launching simulations.

	Conductivity (σ) (S/m)		Relative Permittivity $\left(\varepsilon_{r}\right)$			
			Real Part $\left(\varepsilon^{\prime }\right)$		Imaginary Part $\left(\varepsilon^{\prime \prime }\right)$	
**Frequency (GHz)**	28	60	28	60	28	60
**Material**						
Concrete	0.48	0.89	5.31	5.31	0.31	0.26
Brick	0.03	0.03	3.75	3.75	0.02	0.01
Plasterboard	0.12	0.21	2.94	2.94	0.07	0.06
Wood	0.16	0.37	1.99	1.99	0.10	0.11
Glass	0.22	0.56	6.27	6.27	0.14	0.17
Ceiling board	0.02	0.05	1.5	1.5	0.01	0.01
Chipboard	0.29	0.52	2.58	2.58	0.18	0.15
Floorboard	0.39	1.11	3.66	3.66	0.25	0.33
Metal	10^7^	10^7^	1	1	6.4 × 10^6^	2.9 ×10^6^
Skin tissue	73.22	210.52	28.54	11.97	0	0

**Table 3:** Setting of the main input parameters for 5G and Wi-Fi 802.11ay simulation setup in the considered scenario.

Description	Value	Ref
Number of AP *	9	
AP * antenna configuration	64	[50]
TX power per considered AP * beam	15 dBm	[6,51]
AP * antenna element gain	0 dBi	[6,51]
AP * azimuth beam width	30°	[52]
AP * elevation beam width	30°	[52]
F_TDD_	0.75	[11,53]
UE antenna configuration	**8**	[50]
TX power per considered UE beam	10 dBm	[23]
UE azimuth beam width	65°	[51]
UE elevation beam width	65°	[51]
UE antenna element gain	0 dBi	[6,51]
Carrier frequency 5G/Wi-Fi 802.11ay	28/60 GHz	
3D-RL Angle Resolution	0.4°	
Maximum number of reflections	4	
Maximum number of refractions	1	
Diffraction	Yes	
Maximum number of diffractions/Diffracted ray angular resolution	1/0.25°	
Scenario size	18 × 47 × 7	
Unitary volume analysis	1 m	

* AP: gNodeBs/Wi-Fi 802.11ay.

**Table 4:** Distribution of active users for the different considered cases for the medium user density (MD) and high user density (HD) within the scenario.

		Number of Active Users			
		5G UL	5G DL	802.11ay UL	802.11ay DL
Case I	MD	42	98	-	-
	HD	60	142	-	-
Case II	MD	-	-	42	98
	HD	-	-	60	142
Case III	MD	12	30	29	69
	HD	18	42	42	100

**Table 5:** Measurement transmitter/receiver setup summaries for both frequencies under analysis.

Setup	Equipment	Description
**Transmitter @28 GHz**	Signal generator SMB100A from Rohde & Schwarz, Munich, Germany	Signal generator up to 20 GHz. The transmitted power has been set at 14 dBm
	Frequency multiplier FDA-K/28 from Farran Technologies, Cork, Ireland	Frequency multiplier connected to the signal generator to increase the CW transmitted signal up to 28 GHz
	Ka-band pyramidal horn antenna, model SAR-2013-28KF-E2 from SAGE Millimeter, Inc.	The antenna offers 20 dBi nominal gain and a typical half power beamwidth of 14 degrees on the E-plane and 16 degrees on the H-plane
**Receiver @28 GHz**	Spectrum analyzer N9952A 50 GHZ FieldFox from Keysight Technologies, Santa Rosa, CA, USA	Portable spectrum analyzer up to 50 GHz (see Figure 6 for reference)
	Ka-band omnidirectional antenna Model SAO-2734033045-KF-C1-BL from SAGE Millimeter, Inc.	The antenna model is equipped with a low noise amplifier (LNA) of 30 dBi
**Transmitter @60 GHz**	Signal generator SMB100A from Rohde & Schwarz	Signal generator up to 20 GHz. The transmitted power has been set at 5 dBm
	Frequency multiplier FES-12 from Farran Technologies	Frequency multiplier connected to the signal generator to increase the CW transmitted signal up to 60 GHz
	E-band pyramidal horn antenna	23 dBi nominal gain and a typical half power beamwidth of 10 degrees on the E-plane and 11 degrees on the H-plane
**Receiver @60 GHz**	Spectrum analyzer N9952A 50 GHZ FieldFox from Keysight Technologies	Portable spectrum analyzer up to 50 GHz (see Figure 7 for reference)
	WR-12 Harmonic Mixer Module from OML Inc., Morgan Hill, CA, USA	The Harmonic Mixer Module is designed specifically for handheld spectrum analyzers (see Figure 7 for reference)
	E-band pyramidal horn antenna	23 dBi nominal gain and a typical half power beamwidth of 10 degrees on the E-plane and 11 degrees on the H-plane

**Table 6:** Differences between the measurements and simulation for the different linear distribution radials presented in Figure 14 and Figure 15 and the measurements points of Figure 16.

	Difference Sim. vs. Meas. (dB)	
Frequency	28 GHz	60 GHz
Radials Figure 14 and Figure 15		
R1	1.52	1.84
R2	1.55	2.18
R3	1.30	1.84
R4	1.48	3.23
R5	1.71	1.40
R6	1.67	3.88
R7	3.59	1.89
R8	2.42	5.09
R9	1.20	2.38
R10	1.51	4.13
R11	1.20	4.50
Measurement Points Figure 16	0.86	2.64
TOTAL Difference (mean)	1.67	2.92
